# Dynamic Analysis of Metabolic Response in Gastric Ulcer (GU) Rats with Electroacupuncture Treatment Using ^1^H NMR-Based Metabolomics

**DOI:** 10.1155/2019/1291427

**Published:** 2019-04-22

**Authors:** Jia-cheng Shen, Lin-yu Lian, Yuan Zhang, Qi-da He, Jiao-long Chen, Long-bin Zhang, Miao-sen Huang, Mi Liu, Lin-chao Qian, Cai-chun Liu, Zong-bao Yang

**Affiliations:** ^1^Cancer Research Center, School of Medicine, Xiamen University, Xiamen 361005, China; ^2^College of Acupuncture and Moxibustion, Fujian University of Traditional Chinese Medicine, Fuzhou 350122, China; ^3^College of Acupuncture and Moxibustion, Hunan University of Traditional Chinese Medicine, Changsha 410208, China

## Abstract

Gastric ulcer (GU), a common digestive disease, has a high incidence and seriously endangers health of human. According to the previous studies, it has been proved that electroacupuncture at acupoints of stomach meridian had a good effect on GU. However, there are few published studies on metabolic response in gastric ulcer (GU) rats with electroacupuncture treatment. Herein, we observed the metabolic profiles in biological samples (stomach, liver, and kidney) of GU rats with electroacupuncture treatment by ^1^H NMR metabolomics combined with pathological examination. The male SD rats were induced by intragastric administration of 70% ethanol after fasting for 24 hours and treated by electroacupuncture at Zusanli (ST36) and Liangmen (ST21) for 1 day, 4 days, or 7 days, respectively. And the conventional histopathological examinations as well as metabolic pathways assays were also performed. We found that GU rats were basically cured after electroacupuncture treatment for 4 days and had a complete recovery after electroacupuncture treatment for 7 days by being modulated comprehensive metabolic changes, involved in the function of neurotransmitters, energy metabolism, cells metabolism, antioxidation, tissue repairing, and other metabolic pathways. These findings may be helpful to facilitate the mechanism elucidating of electroacupuncture treatment on GU.

## 1. Introduction 

Gastric ulcer (GU) is a common clinical disease characterized by rhythmic burning pain in upper abdomen and has s a high incidence rate of 10% [1]. Additionally, GU may bring considerable complications including bleeding, perforation, pyloric obstruction, and canceration in stomach and seriously affects living quality of the people [2]. At present, the modern medical treatment of GU mainly includes drugs of HP eradication and acid suppression as well as protection of gastric mucosa and so on. To a certain extent, these treatments are limited in clinic because of side effects, high cost, and drug resistance, although they indeed have good effects in repairing gastric mucosal lesions [3]. Therefore, a more ideal treatment is urgently needed.

Acupuncture, a complementary and alternative therapy, has been used in China for thousands of years and become increasingly popular in western countries because of its significant effect and few side effects [4]. Electroacupuncture is an improved version of acupuncture which can improve the clinical effect by increasing stimulation by delivering appropriate electrical pulses to needles [5]. And it has been reported that electroacupuncture had a good effect on GU [6, 7]. However, there are few studies on the therapeutic mechanism elucidating of electroacupuncture treatment on GU.

Metabolomics is a systems biology approach. It can determine any metabolic changes in biofluids and organism caused by disorders and stimulations in the holistic context, which coincides with the holism of acupuncture [8, 9]. And ^1^H NMR-based metabolomics has been widely used for studies on therapeutic mechanism of electroacupuncture treatment because of its nondestructive sample preparation and nonselective analysis [10–15]. Meanwhile, our previous studies proved that GU can be treated effectively by acupuncture at Liangmen (ST 21) and Zusanli (ST 36) which belong to the stomach meridian [16, 17].

In this study, the therapeutic mechanism of electroacupuncture treatment on GU was investigated by dynamic analysis of metabolic response in gastric ulcer (GU) rats with electroacupuncture treatment using ^1^H NMR-based metabolomics coupled with pathological evaluation.

## 2. Materials and Methods

### 2.1. Materials

The materials included are as follows: 0.25 × 25mm acupuncture needle (Hanyi Medical Instruments Co., Ltd., Beijing, China); Leica paraffin embedding station (EG1150H, Leica Biosystems Nussloch GmbH, Germany); Leica rotary microtome (RM 2235, Leica Biosystems Nussloch GmbH, Germany); upright microscopes (BX53, Olympus Corporation, Japan); deuterium oxide (D2O) and sodium 3-trimethylsilyl-(2,2,3,3-d4)-1-propionate (TSP) (Sigma-Aldrich, Inc., USA); trichloroacetaldehyde hydrate (Sinopharm Chemical Reagent Co. Ltd., Shanghai, China); hematoxylin-eosin solution (Leagene Biotechnology Co., Ltd., Beijing, China).

### 2.2. Animals

54 male healthy male Specified Pathogen Free (SPF) SD rats, weighing 200 ± 20g, were fed in Xiamen University Laboratory Animal Center where temperature was kept at 22-25°C and the relative humidity was controlled at 50% and 12 hours light-dark cycle. In this study, the whole experimental protocol was approved by the Animal Care and Use Committee of Xiamen University (Permit Number: SCXK160803004). “Guide for the Care and Use of Laboratory Animals” of National Institute of Health has been followed in all animals used and care of this study [18].

After 7 days of adaptation, all rats were initially divided into 3 groups (n=18/group) randomly: control group (C), gastric ulcer model group (M), and electroacupuncture group (EA). According to the different intervention courses, rats in each group were randomly divided into 3 groups (n=6/group) as follows: control group (C1), model group (M1), and electroacupuncture group (EA1) for electroacupuncture intervention course of 1 day; control group (C2), model group (M2), and electroacupuncture group (EA2) for electroacupuncture intervention course of 4 days; control group (C3), model group (M3), and electroacupuncture group (EA3) for electroacupuncture intervention course of 7 days. Except for the controls, all rats were modeled gastric ulcer by gavage 70% ethanol after being fasted for 24 hours [19].

### 2.3. Electroacupuncture Treatment

After modeling, the rats in group EA1, EA2, and EA3 were treated by electroacupuncture at Liangmen (ST21) and Zusanli (ST36) for 1 day, 4 days, and 7 days, respectively. These two acupoints were selected according to “The Veterinary Acupuncture of China” and Government Channel and Points Standard GB12346-90 of China. During the experiment, rats in EA groups were fixed on the frame for electroacupuncture treatment (30 minutes/day), while the rats in control and model groups do not accept any intervention except for fixing.

### 2.4. Sample Collection

The animals from three subgroups were, respectively, sacrificed anesthetized with isoflurane after electroacupuncture treatment for 1 day, 4 days, and 7 days. Then stomach, liver, and kidney tissues were collected. 1 cm×1cm gastric mucosa of each stomach was cut and put in 4% formaldehyde for fixation for histopathology examination. Other parts of stomach, liver, and kidney sample were stored at −80°C for follow-up index analysis.

### 2.5. Histopathology

After fixation, the gastric mucosa was dehydrated by ascending series of alcohol, embedded, and sliced in paraffin successively, and the histopathology of gastric mucosa was observe by light microscopy after hematoxylin-eosin staining.

### 2.6. ^1^*H* NMR Experiments

All samples of stomach, liver and kidney were weighed 200 ± 10mg and shred on the ice. The tissues were homogenated in 600 mL of CH3OH and 300 mL of H2O and then vortexed for 1 min. After partitioning on ice for 10 min, the samples were centrifuged for 10 min (10000 rpm, 4°C). Next, the upper supernatant was piped into tubes for lyophilization and then mixed with 600 *μ*L D2O comprising sodium 3-trimethylsilyl-(2, 2, 3, 3-d4)-1-propionate (TSP, 1 mM). Finally, 550 *μ*L of mixture was transferred into 5 mm NMR tube for NMR analysis. And then, a Bruker 600 MHz spectrometer at 298 K was used to obtain ^1^H NMR spectra of these samples. And a Nuclear Overhauser Effect Spectroscopy (NOESY, RD-901-t1-90°-tm-90° -acquire) pulse sequence was used to capture the standard 1D ^1^H spectra; 64 FIDs were collected into 64 K data points with spectral width of 12000 Hz and a relaxation delay of 6.5 *μ*s.

NMR spectra were collected by Bruker NMR spectrometer (600 MHz), and the metabolites in NMR spectra of all samples were identified by published literatures, our own developed NMR database, and chemical shift database of compounds such as BMRB (http://www.bmrb.wisc.edu/metabolomics/) and HMDB (http://www.hmdb.ca/).

The single peak from TSP was referenced at 0.0 ppm, and then all spectra were manual baseline corrected and phased by MestReNova version 9.0.1 software (Mestrelab Research, Santiago de Compostella, Spain). The chemical shift range of 4.70-5.00 ppm was cut in all spectra to remove the influence caused by water. The spectrum was piecewise integral in the region of 0.5-9.0ppm with the width of 0.01ppm. In order to avoid the effect of concentration difference between samples, the integral values of each spectrum were normalized. And multivariate analysis was carried out in SIMCA-P14.1 software (Umetrics, Sweden). Before multivariable analysis, Pareto-scaling (Par) was used to reduce the effect of artifact and noise in the model. Firstly, the principal component analysis (PCA) was used to evaluate the natural separation by visual observation in each group. Then, orthogonal partial least squares discriminant analysis (OPLS-DA) was used to maximize the difference between groups. At the same time, the quality of OPLS-DA model was evaluated by analyzing model fitting (R2) and prediction ability (Q2), and the potential variables were analyzed by the corresponding S-plot of OPLS-DA. Finally, the differential metabolites were obtained according to the variable importance in the project (VIP≥1.00) and the independent-sample t-test (p<0.05) through the OriginProver 8.1.

## 3. Results

### 3.1. Histopathology Examinations

**Figure 1:**
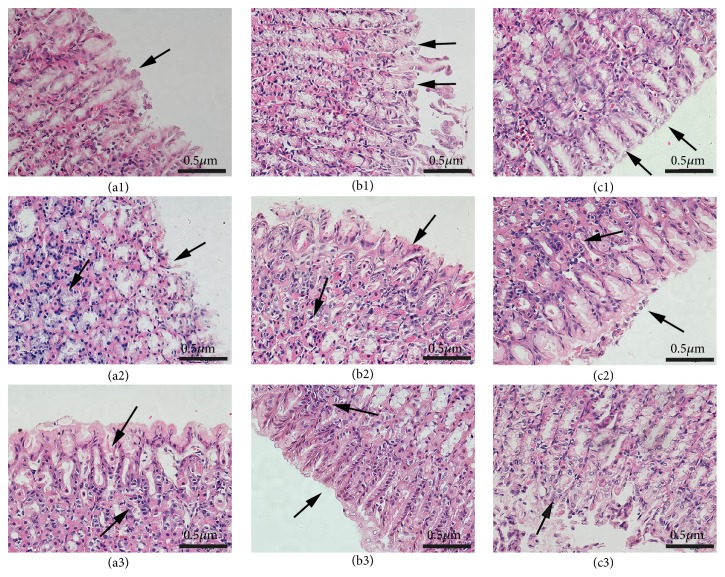
Histological examination of gastric mucosa from all groups ((a1), (a2), and (a3), rats in control group, GU model group, and electroacupuncture at 1 day; (b1), (b2), and (b3), rats in control group, GU model group, and electroacupuncture at 4 days; (c1), (c2), and (c3), rats in control group, GU model group, and electroacupuncture at 7 days). Scale bars represent 0.5 *μ*m in each group.

The results of pathological examination were shown in Figure 1 and Figure S1. The epitheliums of gastric mucosa of control rats were integrity; epithelial cells were marshalling. The mucosa and muscularis were normal; gland was closely arranged, and there are no congestion and edema (in Figures 1(a1), 1(b1), and 1(c1) and Figure S1 a1, b1, and c1). For rats in group M1, the structure of the gastric mucosa was destroyed; the gland was disorganized with a large number of infiltrated inflammatory cells (in Figure 1(a2) and Figure S1 a2). This indicates a successful GU modeling. In Figure 1(a3) and Figure S1 a3, it is showed that the structure of the gastric mucosa was slightly improved, the glands were still disorder, and inflammatory cells were still infiltrated in EA1 group. The gastric mucosa of the rats in M2 group still showed a state of ulcer injury with a large number of inflammatory cells (in Figure 1(b2) and Figure S1 b2), while the rats in EA2 group showed that structure of the gastric mucosa was improved, the gland was clearer, and a small amount of inflammatory cells was infiltrated (in Figure 1(b3) and Figure S1 b3), suggesting that the GU rats were partly improved after electroacupuncture treatment for 4 days. The gastric mucosa of rats in M3 group was relieved than before, but there was still a small amount of inflammatory cell infiltration (in Figure 1(c2) and Figure S1 c2). This may be caused by some self-restoration abilities of rats. Noticeably, for rats in EA3 group (in Figure 1(c3) and Figure S1 c3), the complete structure of the gastric mucosa and clear gland was neatly arranged with no inflammatory cell infiltration, which indicates that GU rats were completely cured after electroacupuncture treatment for 7 days.

### 3.2. ^1^*H* NMR Experiments of Stomach, Liver, and Kidney

**Figure 2:**
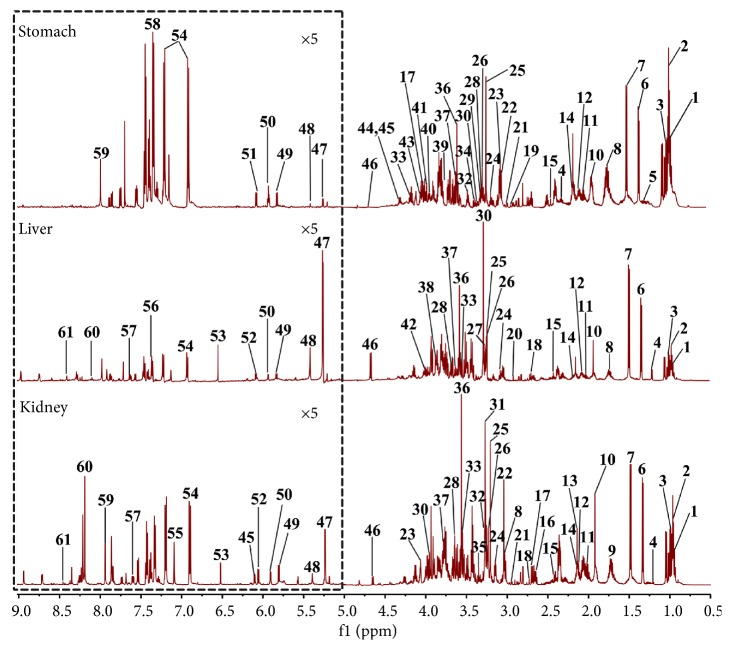
The typical ^1^H NMR spectra of stomach, liver, and kidney of rat. (1, isoleucine; 2, leucine; 3, valine; 4, 3-hydroxybutyrate; 5, methylmalonate; 6, lactate; 7, alanine; 8, lysine; 9, ornithine; 10, acetate; 11, glutamate; 12, glutamine; 13, methionine; 14, glutathione; 15, succinate; 16, citrate(M); 17, aspartate; 18, dimethylamine; 19, methylguanidine; 20, N-methylhydantoin; 21, asparagine; 22, creatine; 23, creatinine; 24, ethanolamine; 25, choline; 26, phosphocholine; 27, phosphoethanolamine; 28, glycerophosphocholine; 29, acetylcholine; 30, betaine; 31, trimethylamine-N-oxide; 32, taurine; 33, inositol; 34, methanol; 35, scyllo-inositol; 36, glycine; 37, glycerol; 38, glycogen; 39, N, N-dimethylglycine; 40, serine; 41, phosphocreatine; 42, glucaric acid; 43, adenosine monophosphate; 44, inosine; 45, adenosine; 46, *β*-glucose; 47, *α*-glucose; 48, allantoin; 49, uracil; 50, uridine; 51, NADP+; 52, cytidine; 53, fumarate; 54, tyrosine; 55, histidine; 56, tryptophan; 57, nicotinamide; 58, phenylalanine; 59, xanthine; 60, hypoxanthine; 61, formate).

The typical ^1^H NMR spectra for extract of stomach, liver, and kidney of rats were shown in Figure 2. 61 endogenous metabolites were found and the related chemical signals were shown in Tables S1–S3.

**Figure 3:**
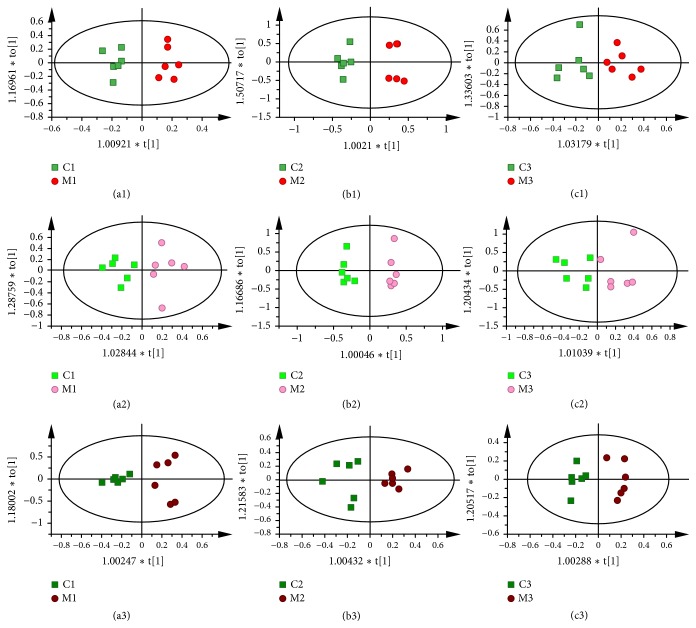
OPLS-DA scores plots from stomach of rats in C1 and M1 group ((a1) R2X=0.39cum, R2Y=0.947cum, and Q2=0.733cum); stomach of rats in C2 and M2 group ((b1) R2X=0.831cum, R2Y=0.962cum, and Q2=0.539cum); stomach of rats in C3 and M3 group ((c1) R2X=0.499cum, R2Y=0.797cum, and Q2=0.379cum); liver of rats in C1 and M1 group ((a2) R2X=0.583cum, R2Y=0.832cum, and Q2=0.575cum); liver of rats in C2 and M2 group ((b2) R2X=0.888cum, R2Y=0.98cum, and Q2=0.819cum); liver of rats in C3 and M3 group ((c2) R2X=0.777cum, R2Y=0.744cum, and Q2=0.528cum); kidney of rats in C1 and M1 group ((a3) R2X=0.789cum, R2Y=0.9cum, and Q2=0.823cum); kidney of rats in C2 and M2 group ((b3) R2X=0.688cum, R2Y=0.862cum, and Q2=0.66cum); kidney of rats in C3 and M3 group ((c3) R2X=0.545cum, R2Y=0.926cum, and Q2=0.786cum).

**Figure 4:**
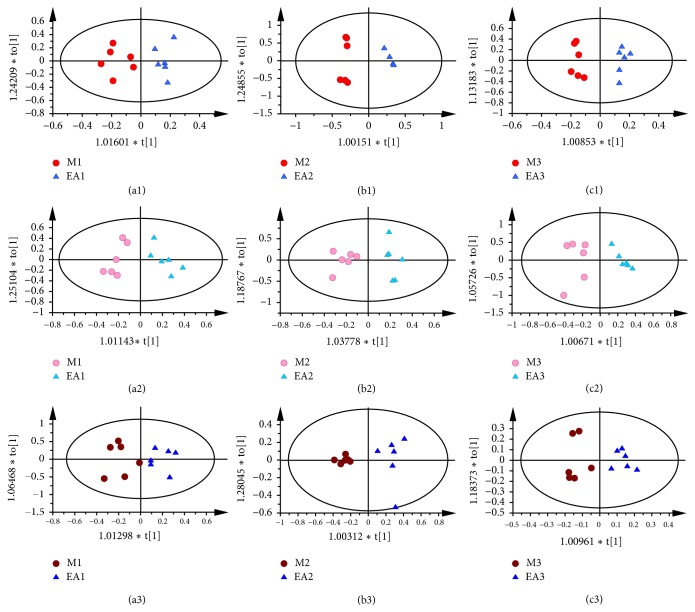
OPLS-DA scores plots from stomach of rats in M1 and EA1 group ((a1) R2X=0.39cum, R2Y=0.868cum, and Q2=0.405cum); stomach of rats in M2 and EA2 group ((b1) R2X=0.798cum, R2Y=0.983cum, and Q2=0.957cum); stomach of rats in M3 and EA3 group ((c1) R2X=0.628cum, R2Y=0.97cum, and Q2=0.541cum); liver of rats in M1 and EA1 group ((a2) R2X=0.483cum, R2Y=0.87cum, and Q2=0.724cum); liver of rats in M2 and EA2 group ((b2) R2X=0.472cum, R2Y=0.919cum, and Q2=0.748cum); liver of rats in M3 and EA3 group ((c2) R2X=0.811cum, R2Y=0.904cum, and Q2=0.779cum); kidney of rats in M1 and EA1 group ((a3) R2X=0.743cum, R2Y=0.804cum, and Q2=0.654cum); kidney of rats in M2 and EA2 group ((b3) R2X=0.707cum, R2Y=0.93cum, and Q2=0.857cum); kidney of rats in M3 and EA3 group ((c3) R2X=0.389cum, R2Y=0.901cum, and Q2=0.71cum).

Visual inspection of ^1^H NMR spectra showed no obvious difference between each group because of the complexity of the spectra. In order to find any possible variables contributing to all groups, the OPLS-DA was subsequently used. As shown in Figure 3, for all sample types, there was a clear separation between GU rats and the controls, indicating that there was a significant metabolic change in the rats with gastric ulcer. Using the same method, EA groups also separated obviously from model groups in Figure 4, suggesting that electroacupuncture treatment had an obvious effect on GML.

In order to further filter the metabolites related to pathology of GU and electroacupuncture treatment, we also performed the corresponding S-plot and t-test (in supplementary Figures S2 and S3). Compared with control groups, the level of metabolites in model groups had some changes as follows: (a) In gastric tissue, the levels of isoleucine, leucine, valine, glutamate, glutamine, glycerol, phenylalanine, and tyrosine decreased, while the levels of taurine and serine increased in rats of M1 group; the levels of glycerol decreased, whereas the levels of isoleucine, leucine, valine, serine, phenylalanine, taurine, and tyrosine increased in rats of M2 group; and there are higher levels of serine and glycerol in rats of M3 group. (b) For liver samples, the levels of leucine, valine, isoleucine, glutamate, succinate, and tyrosine were decreased, while the levels of choline, inositol, glycine, glutamine, glycerol, alanine, and betaine were increased in the rats of M1 group; in rats of M2 group, the levels of glutamine, succinate, taurine, betaine, glycine, and glycerol were increased with decreased choline; the lower levels of isoleucine, leucine, valine, and glutamate but higher levels of glutamine, glycerol, alanine, and betaine occurred in rats of M3 group. (c) In kidney, the levels of isoleucine, leucine, valine, alanine, ornithine, glutamine, methionine, and ethanolamine were decreased, while the levels of glycine, phosphocholine, glycerol, glycerophosphocholine, betaine, creatine, and choline were increased in rats of M1 group. The levels of isoleucine, leucine, alanine, glutamine, methionine, and ethanolamine were decreased, whereas the levels of glycine, phosphocholine, glycerol, glycerophosphocholine, betaine, creatine, and choline were increased in rats of M2 group; Rats in M3 group had the lower levels of glycerol, glycerophosphocholine, betaine, creatine and choline and higher levels of leucine, valine, alanine, ornithine, phosphocholine and glutamine. The relative abundance of potential metabolites was shown in the Supplementary Figures S4–12.

Compared with the gastric ulcer model group, the EA group had the following changes: (a) in the gastric tissue, there was upregulated serine in rats of EA1 group; for rats of EA2 group, the levels of isoleucine, valine, glycerol, and serine were increased; and the increased serine occurred in rats of EA3 group. (b) In liver, there are in increased levels of isoleucine, leucine, valine, and tyrosine, while the levels of alanine, choline, inositol, glycine, glutamine, and glycerol were decreased in rats of EA1 group; for rats of EA2 group, the levels of glycine, glycerol, glutamine, alanine, and betaine were downregulated; and the levels of isoleucine, leucine, valine, glutamate were upregulated, while betaine and glycerol were downregulated in rats of EA3 group. (c) In kidney, the levels of isoleucine, leucine, valine, alanine, ornithine, glutamine, and methionine were increased, while the levels of glycerol, glycerophosphocholine, creatine, and choline were decreased in rats of EA1 group. For rats of EA2 group, the levels of isoleucine, leucine, alanine, methionine, ethanolamine, and glutamine were upregulated, whereas the levels of glycine, phosphocholine, glycerol, glycerophosphocholine, betaine, creatine, and choline were downregulated. And the levels of leucine, valine, glutamine, and alanine were increased, while the levels of glycerol, glycerophosphocholine, and betaine were decreased in EA3 group. The relative abundance of potential metabolites was shown in Supplementary Figures S4–12.

**Figure 5:**
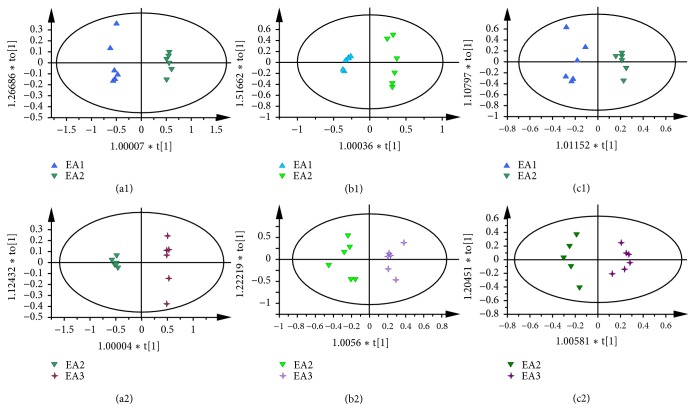
OPLS-DA scores plots from stomach of rats in EA1 and EA2 group ((a1) R2X=0.833cum, R2Y=0.994 cum, and Q2=0.985cum); stomach of rats in EA2 and EA2 group ((a2) R2X=0.802cum, R2Y=0.997cum, and Q2=0.991cum); liver of rats in EA1 and EA2 group ((b1) R2X=0.894cum, R2Y=0.985 cum, and Q2=0.876cum); liver of rats in EA2 and EA3 group ((b2) R2X=0.652cum, R2Y=0.912cum, and Q2=0.76cum); kidney of rats in EA1 and EA2 group ((c1) R2X=0.617cum, R2Y=0.957cum, and Q2=0.82cum); kidney of rats in EA1 and EA2 group ((c2) R2X=0.639cum, R2Y=0.957cum, and Q2=0.816cum).

At the same time, the OPLS-DA was conducted to compare rats with different electroacupuncture courses. For all sample types, there was a clearer separation between EA1 group and EA2 group than EA2 group and EA3 group (in Figure 5), indicating that GU rats have an obvious recovery after electroacupuncture for 4 days. According to the corresponding S-plot and t-test t (in Supplementary Figure S13), during the electroacupuncture treatment, there are some metabolites changes in GU rats as follows: (a) In the gastric tissue, compared with the EA1 group, the levels of isoleucine, leucine, valine, glutamate, glutamine, glycerol, phenylalanine, and tyrosine were increased with decreased alanine and taurine in rats of EA2 group, while there were decreased levels of isoleucine, leucine, valine, glutamine, glycerol, glutamate, phenylalanine, and tyrosine and increased levels of serine and taurine in rats of EA3 group compared with EA2 group. (b) In the liver, the downregulated levels of leucine, valine, isoleucine, glutamate, and succinate were upregulated, whereas alanine, glutamine, betaine, choline, inositol, glycine, and glycerol occurred in rats of EA2 group compared with that of EA1 group, whereas there were decreased levels of leucine, valine, isoleucine, and succinate and increased levels of glycerol and betaine in rats of EA3 group compared with that of EA2 group. (c) In kidney, the levels of methionine, choline, betaine, glutamine, glycerol, and alanine were upregulated in EA2 group compared with the EA1 group, while the rats of EA3 group showed upregulated levels of methionine and choline and downregulated levels of glutamine and betaine compared with rats in EA2 group. The relative abundance of potential metabolites was shown in the supplementary Figures S14–16.

## 4. Discussion

**Figure 6:**
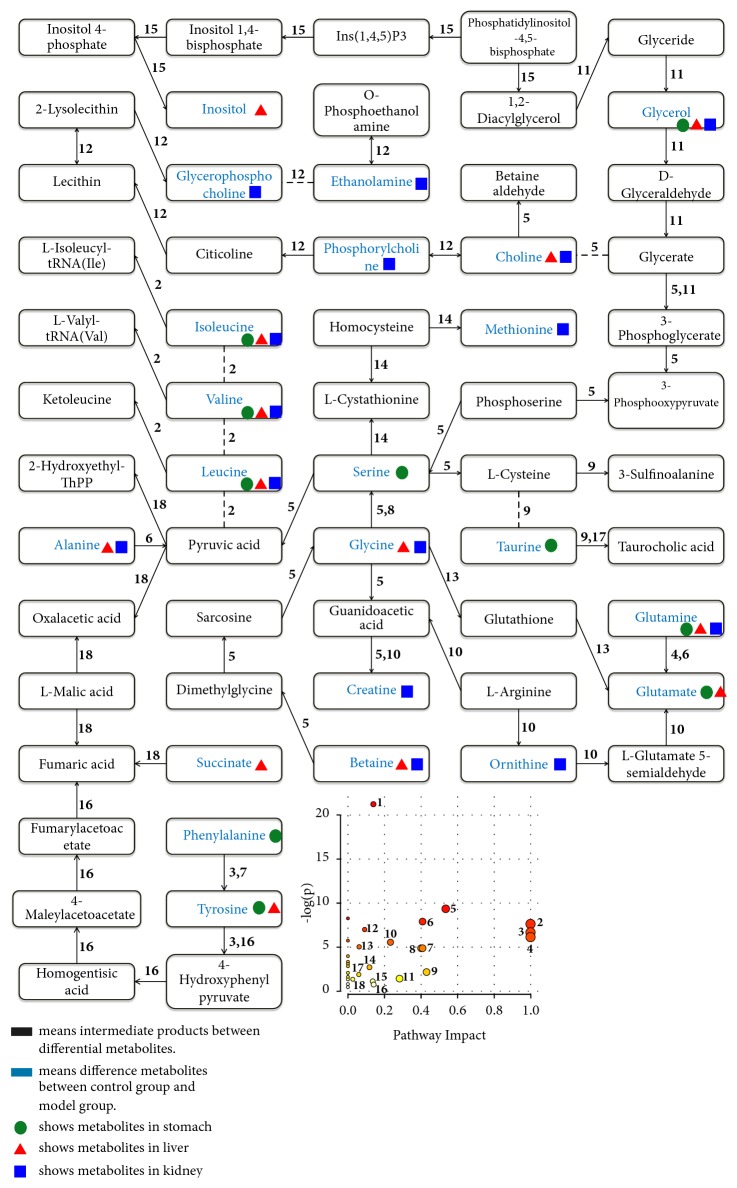
The metabolic pathways related to rats with gastric ulcer and the treatment of electroacupuncture (1, aminoacyl-tRNA biosynthesis; 2, valine, leucine, and isoleucine biosynthesis; 3, phenylalanine, tyrosine, and tryptophan biosynthesis; 4, D-glutamine and D-glutamate metabolism; 5, glycine, serine, and threonine metabolism; 6 alanine, aspartate, and glutamate metabolism; 7, phenylalanine metabolism; 8, methane metabolism; 9, taurine and hypotaurine metabolism; 10, arginine and proline metabolism; 11, glycerolipid metabolism; 12, glycerophospholipid metabolism; 13, glutathione metabolism; 14, cysteine and methionine metabolism; 15, inositol phosphate metabolism; 16, tyrosine metabolism; 17, primary bile acid biosynthesis; 18, citrate cycle (TCA cycle)).

As an important digestive organ of the human body, the stomach is closely related to various related metabolism. Similarly, the liver and kidney are also the important organs of the body and closely involved in various metabolic pathways [20–22]. There are studies showing that gastric ulcer is mainly caused by gastric mucosal injury and affects many metabolic pathways of many organs such as liver and kidney [23]. In Chinese concept, liver and kidney include not only the organs in the views of western medicine, but also other important functions in TCM; the liver has function of regulating qi, the blood, and emotions, while the kidney has impact with bone. So, to some extent, the different levels of changes in metabolites in liver and kidney samples induced by GU which were reversed by electroacupuncture treatment could imply the difference metabolites in liver and kidney in Chinese concept. Recently, acupuncture has attracted much attention with the extensive use of TCM. And both basic research and clinical practice have proved that electroacupuncture had an obvious effect on gastric ulcer, but the therapeutic mechanism has not been very clear yet. Moreover, in our early studies [10, 13], we found that compared with normal rats, the metabolites in tissues (such as stomach, liver, kidney, and brain) and body fluids (such as blood and urine) of gastric diseases rats were changed. After treatment, the metabolites in the above organs and body fluids will be reversed to some extent. Therefore, we believe that diseases can affect the functions of some organs and body fluids and then cause changes in their metabolites, which can be reversed after electroacupuncture. In other words, electroacupuncture can change the related metabolites by repairing the related physiological functions of the body, and we can speculate the mechanism of electroacupuncture on related diseases by observing the changes of related metabolites. In this study, the therapeutic mechanism of electroacupuncture treatment on GU was investigated by a dynamic analysis of metabolomic profiling in biological samples (stomach, liver, and kidney) of GU rats using ^1^H NMR-based metabolomics. We also found considerable metabolites related to GU and electroacupuncture treatment, involved in multiple metabolic pathways (in Figure 6). The details will be further discussed below.

### 4.1. Neurotransmitter Metabolism

Glutamate is the most abundant and fast excitatory neurotransmitter in the nervous system, and as the precursor of glutamate neurotransmitters, glutamine plays an important role in maintaining the integrity of the gastrointestinal mucosa [24–26]. In addition, phenylalanine is also a precursor of tyrosine and monoamine neurotransmitters and can syntheses important neurotransmitters with tyrosine [27]. In this study, the levels of tyrosine and glutamine in liver of GU rats were reversed by electroacupuncture treatment for 1 day, while the levels of tyrosine and phenylalanine in gastric tissue of GU rats returned to normal after electroacupuncture treatment for 4 days. And the level of glutamate in liver tissue of GU rats was also reached the normal after electroacupuncture treatment for 7 days. These proved that electroacupuncture treatment may have a good effect on GU by regulating the function of neurotransmitters.

### 4.2. Energy Metabolism

Glycerol, the skeleton of fatty acids in fat, provides energy for cells by converting to glucose [28]. Alanine also is a kind of energy and participates in the glucose-alanine cycle. During this cycle, amino acids are decomposed and collected in the form of glutamate and alanine in muscles and other tissues of mammals by alanine aminotransferase [29]. In addition, succinate plays an important role in three carboxylic acid (TCA) cycle and participates in the synthesis of adenosine triphosphate (ATP) [30]. On the other hand, the main role of creatine is to promote the recycling of ATP by providing phosphoric acid groups to adenosine diphosphate (ADP) and transforming it into ATP [31]. In this study, the levels of glycerol and alanine in liver of GU rats were reversed to normal after electroacupuncture treatment for 1 day; the renal levels of glycerol, creatine, and alanine in GU rats reached the normal after electroacupuncture treatment for 4 days. Additionally, the level of glycerol in the gastric tissue of GU rats was reversed to normal after electroacupuncture treatment for 7 days. This indicates that electroacupuncture treatment plays an important role in regulating the energy metabolism of GU rats.

### 4.3. Cell Metabolism

Inositol acts, as a second messenger in the intracellular signal transduction pathway, participate in the cytoskeletal assembly and maintenance of cell membrane potential [32]. Betaine, existing in cells, maintains protein structure and integrity of cell membrane and retains water in cells as an osmotic regulator [33]. Choline is the main source of betaine synthesis and also maintains the integrity of cell membrane and signal transmission as an important component of cell membrane, [34, 35]. Phosphocholine is converted from choline and further synthesized into phosphatidylcholine (lecithin), which is an important component of cell membrane [36, 37]. Glycerophosphocholine can be obtained by the decomposition of lecithin. And it is an intracellular osmotic regulator and its content depends on the uptake of inositol [38]. Ethanolamine is an important component of lecithin and related to the composition of cell membrane [39]. Taurine has the functions of antioxidation, stabilizing cell membrane, regulating cell osmotic pressure, and signaling regulation [40]. In this study, all of these metabolites are significantly changed compared with the controls, suggesting that there was an obvious disorder of cell metabolism in GU rats. On the other hand, the levels of choline and inositol in liver and betaine, phosphocholine, and glycerophosphocholine in kidney were reversed after electroacupuncture treatment for 1 day. Additionally, the level of ethanolamine in kidney was return to normal after electroacupuncture treatment for 4 days and the level of taurine restored to normal after electroacupuncture treatment for 7 days. These findings indicate that electroacupuncture treatment on GU is related to regulating the cell metabolism.

### 4.4. Other Metabolisms

Isoleucine, leucine, and valine, branched chain amino acids, are the essential amino acids of human body and repair tissue and provide energy [41]. Glycine, a precursor to many proteins, is also an antioxidant and an amino acid of endogenous antioxidant glutathione [42]. As a precursor of many kinds of amino acids, serine is involved in the synthesis of amino acids and plays an important role in the catalytic function of many enzymes [43]. Methionine is involved in the synthesis of proteins and inhibits the damage caused by excessive oxidation of cell membrane by oxygen free radicals in the body through various way as an antioxidant [44]. Ornithine is the central substance in the urea cycle and used to treat excess nitrogen [45]. Increased serine in the gastric mucosa of GU rats suggest that gastric ulcer inhibits the synthesis of substances in the stomach and the catalysis of enzymes, while its downregulation after electroacupuncture treatment for 1 day proves that electroacupuncture treatment has an obvious effect in regulating catalysis of enzymes in stomach of GU rats. Additionally, increased methionine and glycine in kidney and glycine in liver of GU rats indicates that GU seriously impacts oxidative stress in liver and kidney and inhibits the peroxidation and protein synthesis in kidney. These two metabolites returned to normal after electroacupuncture treatment, suggesting that electroacupuncture treats GU by regulating the oxidative stress and protein synthesis. On the other hand, the abnormal of isoleucine, leucine, and valine reversed by electroacupuncture treatment for 4 days suggests that electroacupuncture treatment can effectively repair tissues.

To sum up, these findings suggests electroacupuncture treatment can effectively cure GU by regulating some metabolites related to GU, involved in neurotransmitter metabolism, energy metabolism, cell metabolism, antioxidant, tissue repairing, and so on. According to the dynamic analysis of metabolomic profiling in biological samples (stomach, liver, and kidney) of GU rats and pathological examination, the gastric mucosal injury of GU rats was improved effectively after electroacupuncture treatment for 4 days and the curative effect tended to be stable after 7 days of electroacupuncture treatment, which will be beneficial for better understanding the time-relative therapeutic mechanism of on electroacupuncture treatment on GU. And we will also perform the studies research on in-depth molecular response of electroacupuncture treatment on GU in our further study.

## Data Availability

The data used to support the findings of this study are available from the corresponding author upon request.

## Supplementary Materials

Supplementary Materials Figure S1: histological examination of gastric mucosa from all groups (a1, a2, and a3, rats in control group, GU model group, and electroacupuncture at 1 day; b1, b2, and b3, rats in control group, GU model group, and electroacupuncture at 4 days; c1, c2, and c3, rats in control group, GU model group and electroacupuncture at 7 days). Scale bars represent 2 *μ*m in each group. Figure S2: corresponding S-plots from stomach of rats in C1 and M1 group (A); stomach of rats in C2 and M2 group (B1); stomach of rats in C3 and M3 group (C1); liver of rats in C1 and M1 group (A2); liver of rats in C2 and M2 group (B2); liver of rats in C3 and M3 group (C2); kidney of rats in C1 and M1 group (A3); kidney of rats in C2 and M2 group (B3); kidney of rats in C3 and M3 group (C3). Figure S3: corresponding S-plots from stomach of rats in M1 and EA1 group (A1); stomach of rats in M2 and EA2 group (B1); stomach of rats in M3 and EA3 group (C1); liver of rats in M1 and EA1 group (A2); liver of rats in M2 and EA2 group (B2); liver of rats in M3 and EA3 group (C2); kidney of rats in M1 and EA1 group (A3); kidney of rats in M2 and EA2 group (B3); kidney of rats in M3 and EA3 group (C3). Figure S4: relative abundance (mean ± SD) of characteristic metabolites from gastric tissues of rats in C1, M1, and EA1 group. Figure S5: relative abundance (mean ± SD) of characteristic metabolites from gastric tissues of rats in C2, M2, and EA2 group. Figure S6: relative abundance (mean ± SD) of characteristic metabolites from gastric tissues of rats in C3, M3, and EA3 group. Figure S7: relative abundance (mean ± SD) of characteristic metabolites from liver tissues of rats in C1, M1, and EA1 group. Figure S8: relative abundance (mean ± SD) of characteristic metabolites from liver tissues of rats in C2, M2, and EA2 group. Figure S9: relative abundance (mean ± SD) of characteristic metabolites from liver tissues of rats in C3, M3, and EA3 group. Figure S10: relative abundance (mean ± SD) of characteristic metabolites from kidney tissues of rats in C1, M1, and EA1 group. Figure S11: relative abundance (mean ± SD) of characteristic metabolites from kidney tissues of rats in C2, M2, and EA2 group. Figure S12: relative abundance (mean ± SD) of characteristic metabolites from kidney tissues of rats in C3, M3, and EA3 group. Figure S13: corresponding S-plots from stomach of rats in M1 and EA1 group (A); stomach of rats in M2 and EA2 group (B1); stomach of rats in M3 and EA3 group (C1); liver of rats in M1 and EA1 group (A2); liver of rats in M2 and EA2 group (B2); liver of rats in M3 and EA3 group (C2); kidney of rats in M1 and EA1 group (A3); kidney of rats in M2 and EA2 group (B3); kidney of rats in M3 and EA3 group (C3). Figure S14: relative abundance (mean ± SD) of characteristic metabolites from gastric tissues of rats in EA1, EA2, and EA3 group. Figure S15: relative abundance (mean ± SD) of characteristic metabolites from liver tissues of rats in EA1, EA2, and EA3 group. Figure S16: relative abundance (mean ± SD) of characteristic metabolites from kidney tissues of rats in EA1, EA2, and EA3 group. Table S1: peak attribution of the main marked metabolites in 1H-NMR spectra of stomach sample. Table S2: peak attribution of the main marked metabolites in 1H-NMR spectra of liver sample. Table S3: peak attribution of the main marked metabolites in 1H-NMR spectra of kidney sample. Table S4: relative content in control, model, and treatment group. Table S5: relative content in different treatment group over time. Table S6: p (CV-ANOVA) indicating the model quality of OPLS-DA.
