# Supplementary material for: Dynamic Analysis of Metabolic Response in Gastric Ulcer (GU) Rats with Electroacupuncture Treatment Using 1H NMR-Based Metabolomics
Source: Evid Based Complement Alternat Med. 2019 Apr 22;2019:1291427. doi: 10.1155/2019/1291427 (PMC6501414; doi:10.1155/2019/1291427)
Supplement: Supplementary Materials — Figure S1: histological examination of gastric mucosa from all groups (a1, a2, and a3, rats in control group, GU model group, and electroacupuncture at 1 day; b1, b2, and b3, rats in control group, GU model group, and electroacupuncture at 4 days; c1, c2, and c3, rats in control group, GU model group and electroacupuncture at 7 days). Scale bars represent 2 μm in each group. Figure S2: corresponding S-plots from stomach of rats in C1 and M1 group (A); stomach of rats in C2 and M2 group (B1); stomach of rats in C3 and M3 group (C1); liver of rats in C1 and M1 group (A2); liver of rats in C2 and M2 group (B2); liver of rats in C3 and M3 group (C2); kidney of rats in C1 and M1 group (A3); kidney of rats in C2 and M2 group (B3); kidney of rats in C3 and M3 group (C3). Figure S3: corresponding S-plots from stomach of rats in M1 and EA1 group (A1); stomach of rats in M2 and EA2 group (B1); stomach of rats in M3 and EA3 group (C1); liver of rats in M1 and EA1 group (A2); liver of rats in M2 and EA2 group (B2); liver of rats in M3 and EA3 group (C2); kidney of rats in M1 and EA1 group (A3); kidney of rats in M2 and EA2 group (B3); kidney of rats in M3 and EA3 group (C3). Figure S4: relative abundance (mean ± SD) of characteristic metabolites from gastric tissues of rats in C1, M1, and EA1 group. Figure S5: relative abundance (mean ± SD) of characteristic metabolites from gastric tissues of rats in C2, M2, and EA2 group. Figure S6: relative abundance (mean ± SD) of characteristic metabolites from gastric tissues of rats in C3, M3, and EA3 group. Figure S7: relative abundance (mean ± SD) of characteristic metabolites from liver tissues of rats in C1, M1, and EA1 group. Figure S8: relative abundance (mean ± SD) of characteristic metabolites from liver tissues of rats in C2, M2, and EA2 group. Figure S9: relative abundance (mean ± SD) of characteristic metabolites from liver tissues of rats in C3, M3, and EA3 group. Figure S10: relative abundance (mean ± SD) of characteristic m [file 1291427.f1.pdf]

Dynamic analysis of metabolic response in gastric ulcer (GU) rats with electro-acupuncture treatment using ^{1}H NMR-based metabolomics

Jia-cheng Shen[1], Lin-yu Lian[1,2]†, Yuan Zhang[1,3], Qi-da He[1,2], Jiao-long Chen[1,2], Long-bin Zhang[1], Miao-sen Huang[1,2], Mi Liu[3], Lin-chao Qian[1*], Cai-chun Liu[1*], Zong-bao Yang[1*]

[1]Department of Traditional Chinese Medicine and Shenzhen Research Institute, Xiamen University, Xiamen 361005, China

[2] College of Acupuncture and Moxibustion, Fujian university of Traditional Chinese Medicine, Fuzhou 350122, China

[3] College of Acupuncture and Moxibustion, Hunan university of Traditional Chinese Medicine, Changsha 410208, China

Correspondence should be addressed to Zong-bao Yang, yzbldq@163.com and Caichun Liu, caichunliu714@gmail.com.

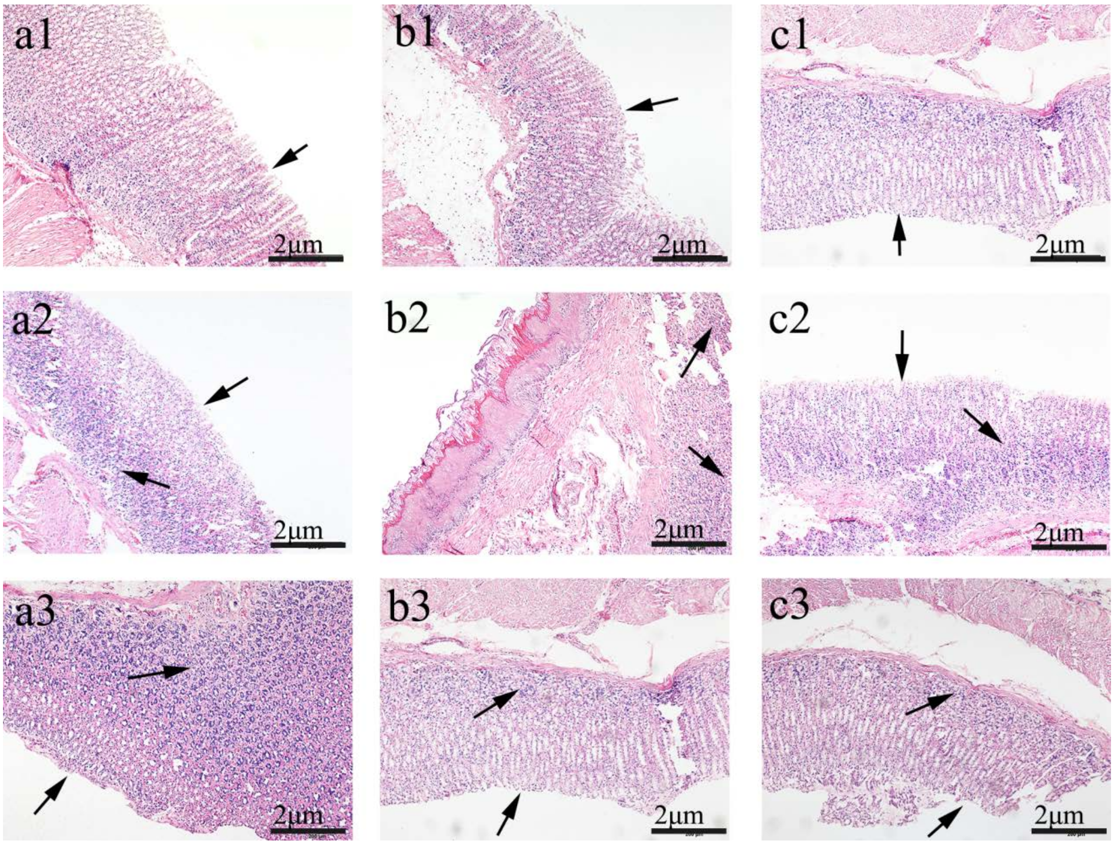

Fig.S1 Histological examination of gastric mucosa from all groups.(a1, a2 and a3, rats in control group, GU model group and electro-acupuncture at 1day; b1, b2 and b3, rats in control group, GU model group and electro-acupuncture at 4days; c1, c2 and c3, rats in control group, GU model

group and electro-acupuncture at 7days). Scale bars represent 2 μm in each group.

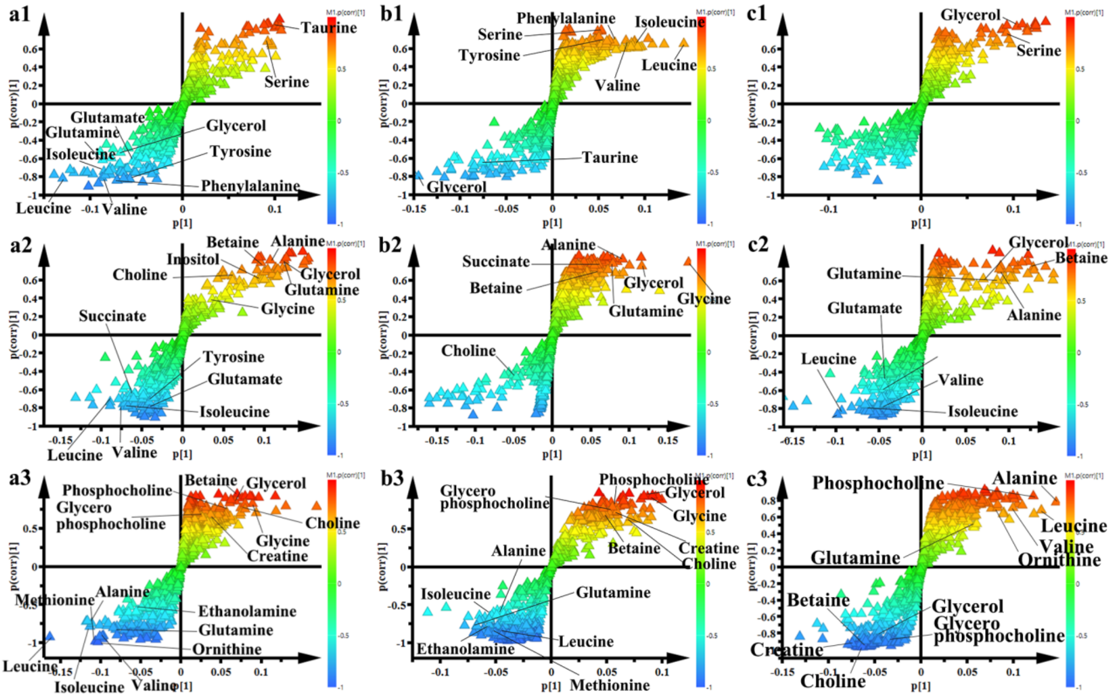

Fig.S2 Corresponding S-plots from stomach of rats in C1 and M1 group (A); stomach of rats in C2 and M2 group (B1); stomach of rats in C3 and M3 group (C1); liver of rats in C1 and M1 group (A2); liver of rats in C2 and M2 group (B2); liver of rats in C3 and M3 group (C2); kidney of rats in C1 and M1 group (A3); kidney of rats in C2 and M2 group (B3), kidney of rats in C3 and M3 group (C3).

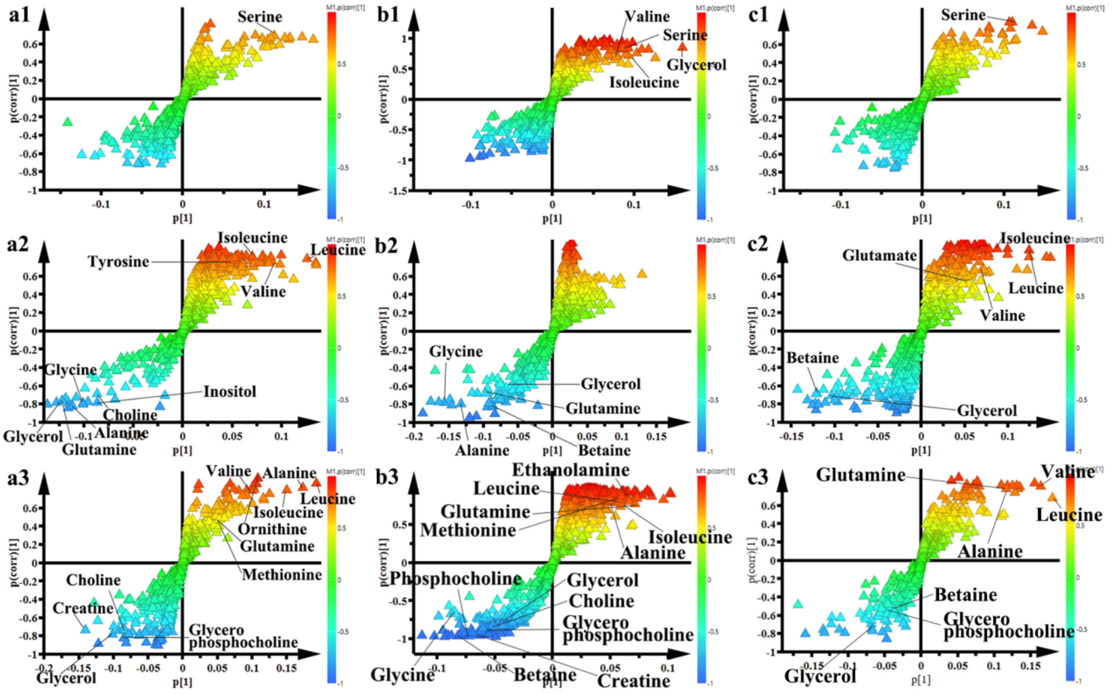

Fig.S3 Corresponding S-plots from stomach of rats in M1 and EA1 group (A1); stomach of rats in

M2 and EA2 group (B1); stomach of rats in M3 and EA3 group (C1); liver of rats in M1 and EA1 group (A2); liver of rats in M2 and EA2 group (B2); liver of rats in M3 and EA3 group (C2); kidney of rats in M1 and EA1 group (A3); kidney of rats in M2 and EA2 group (B3); kidney of rats in M3 and EA3 group (C3).

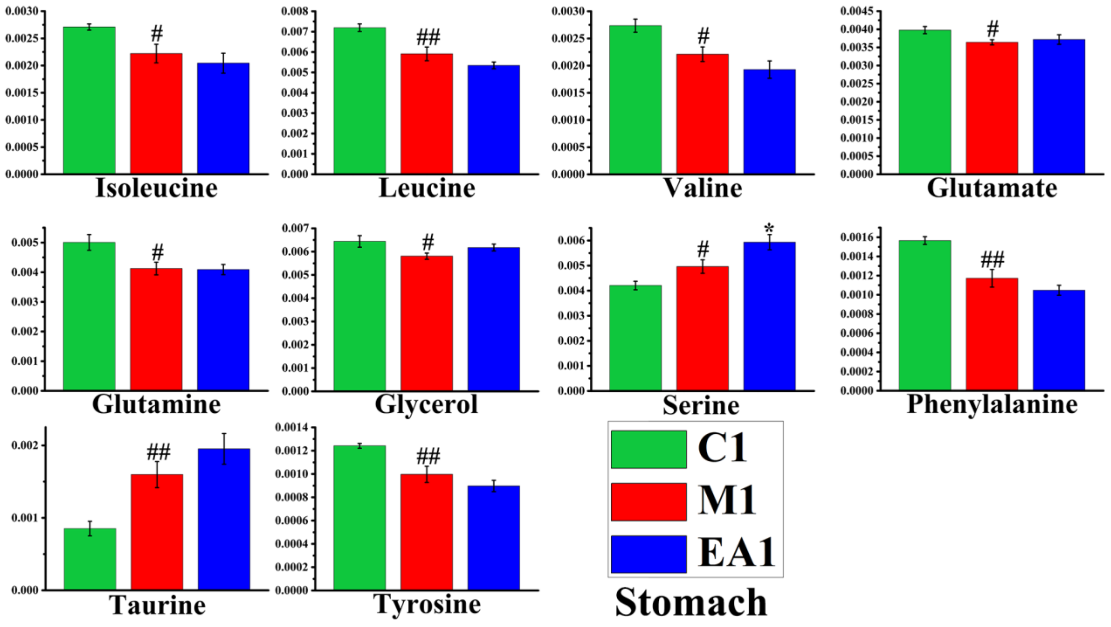

Fig.S4 Relative abundance (mean ± S.D.) of characteristic metabolites from gastric tissues of rats in C1, M1 and EA1 group.

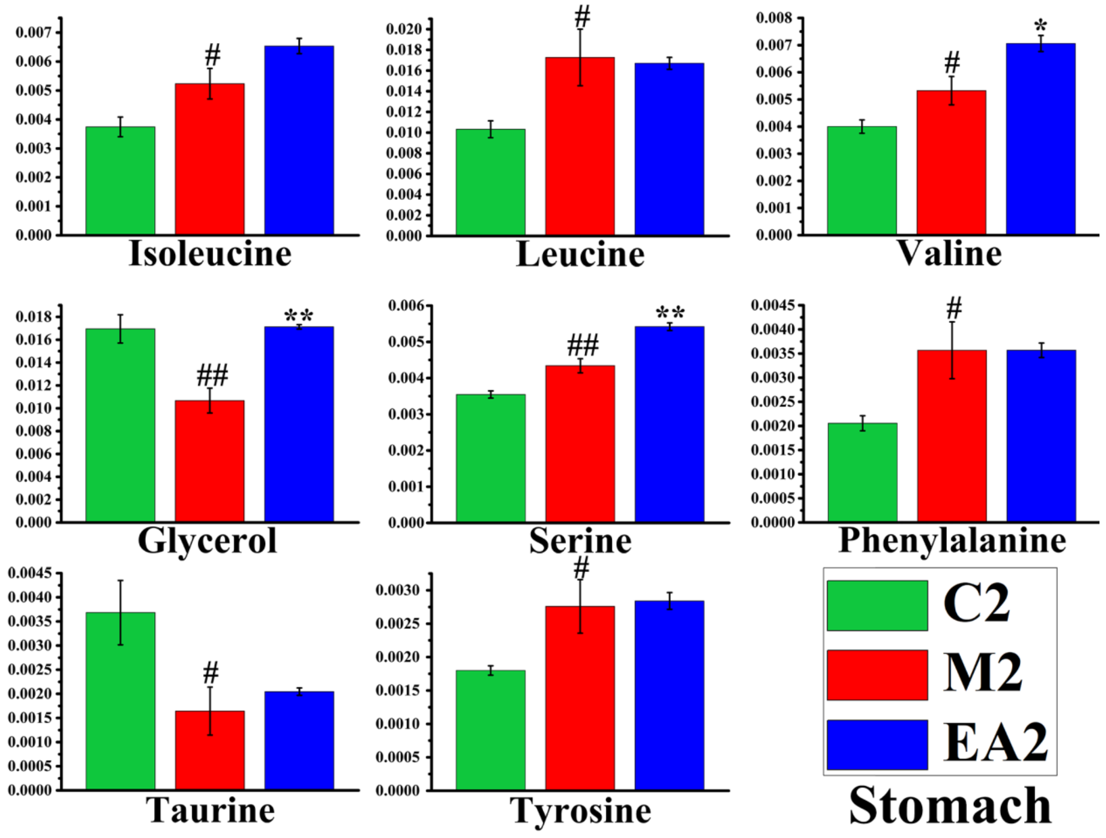

Fig.S5 Relative abundance (mean ± S.D.) of characteristic metabolites from gastric tissues of rats in C2, M2 and EA2 group.

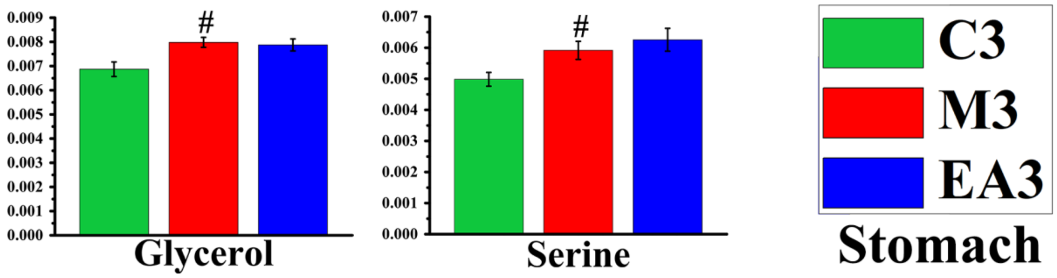

Fig.S6 Relative abundance (mean ± S.D.) of characteristic metabolites from gastric tissues of rats in C3, M3 and EA3 group..

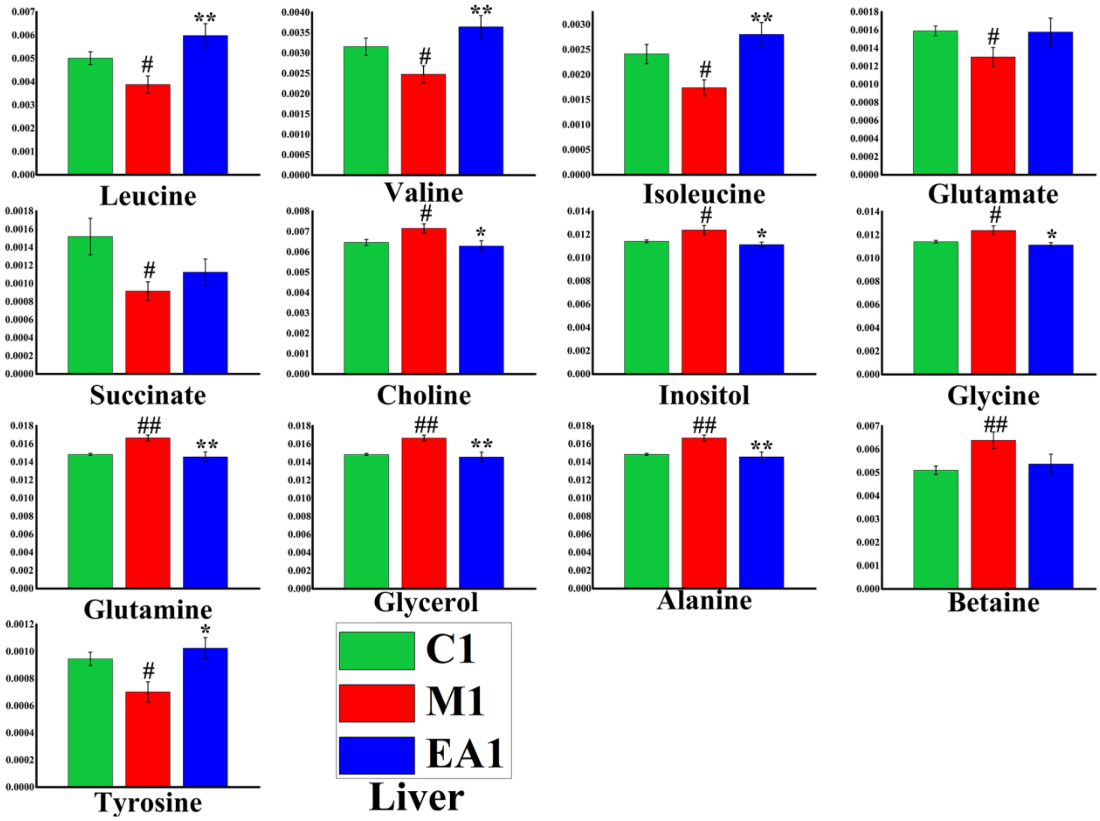

Fig.S7 Relative abundance (mean ± S.D.) of characteristic metabolites from liver tissues of rats in C1, M1 and EA1 group.

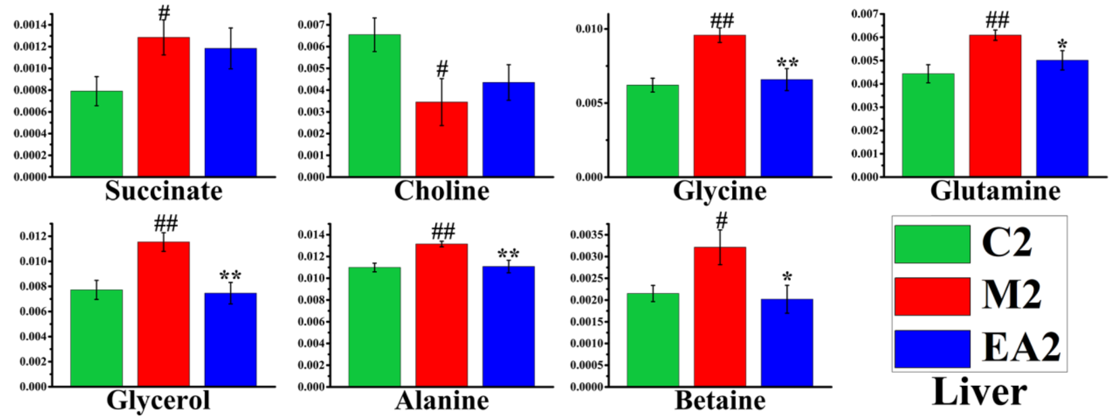

Fig.S8 Relative abundance (mean ± S.D.) of characteristic metabolites from liver tissues of rats in C2, M2 and EA2 group.

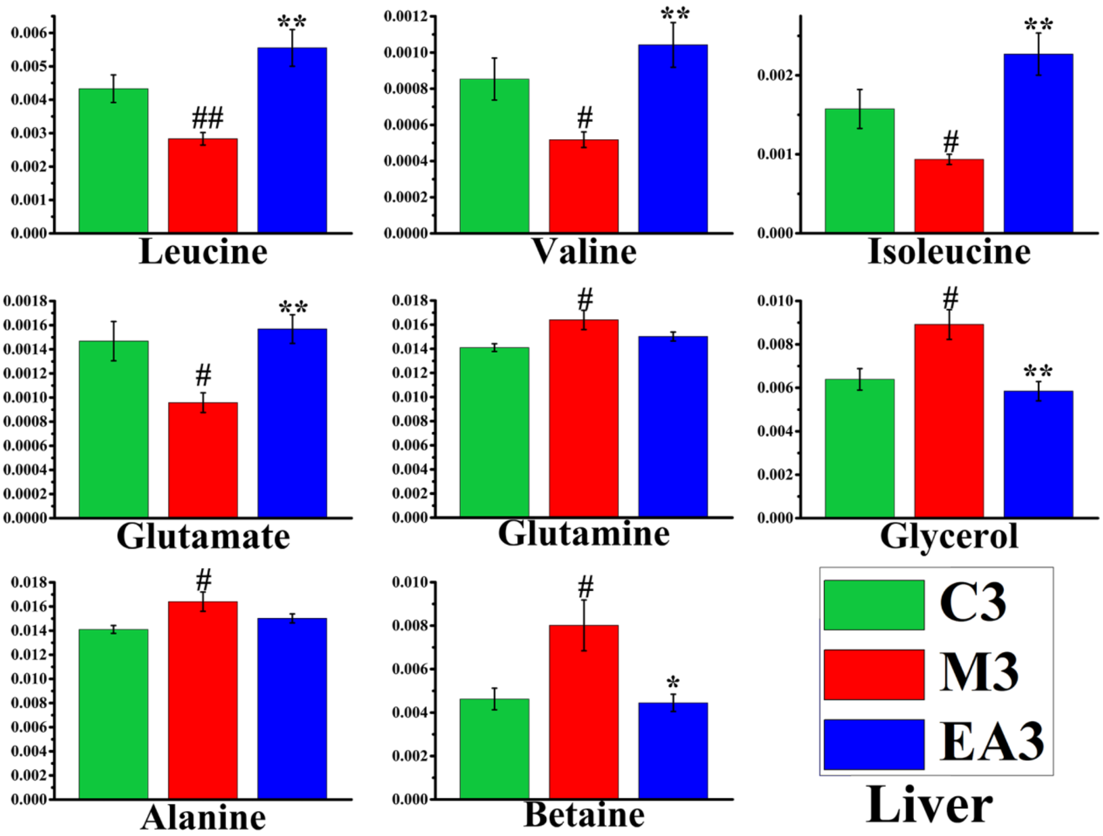

Fig.S9 Relative abundance (mean ± S.D.) of characteristic metabolites from liver tissues of rats in C3, M3 and EA3 group.

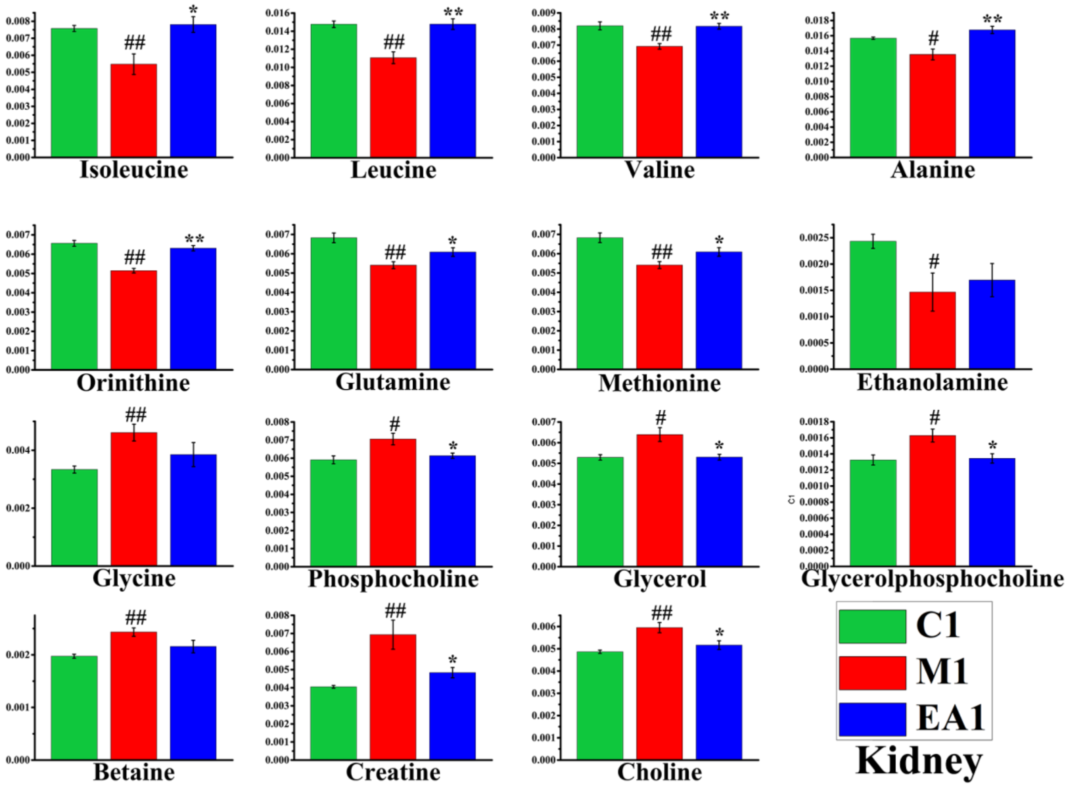

Fig.S10 Relative abundance (mean ± S.D.) of characteristic metabolites from kidney tissues of rats in C1, M1 and EA1 group.

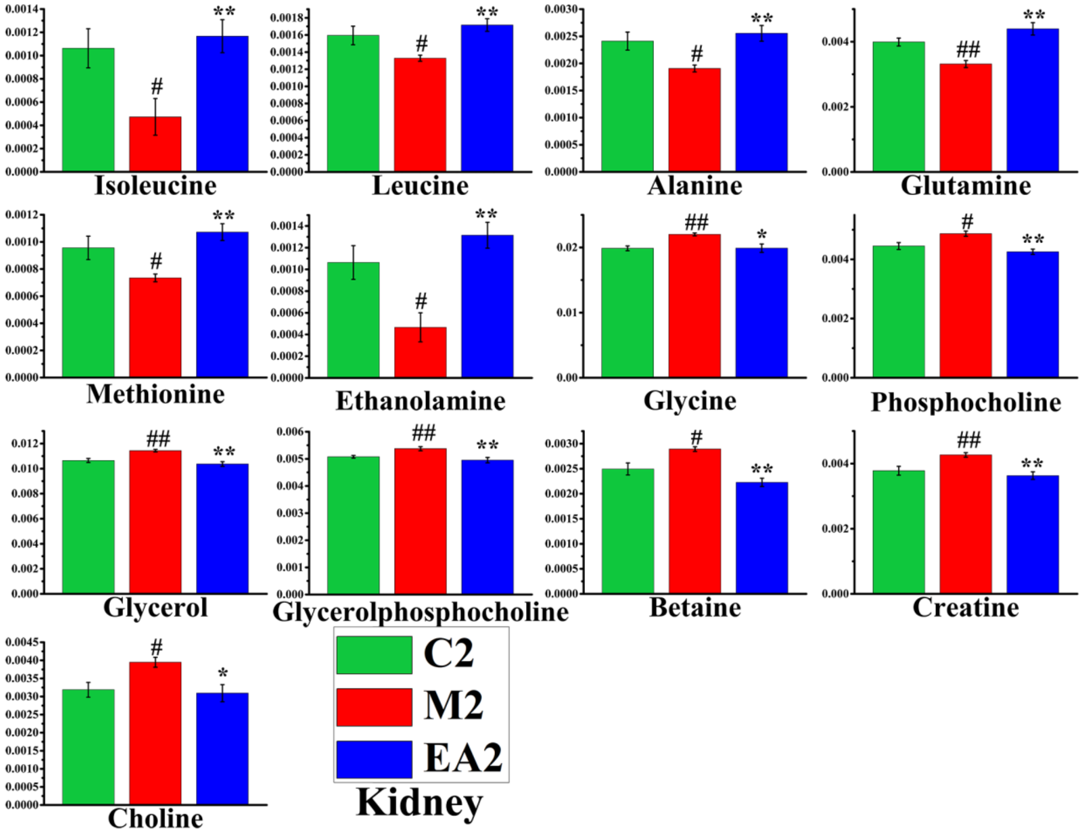

Fig.S11 Relative abundance (mean ± S.D.) of characteristic metabolites from kidney tissues of rats in C2, M2 and EA2 group.

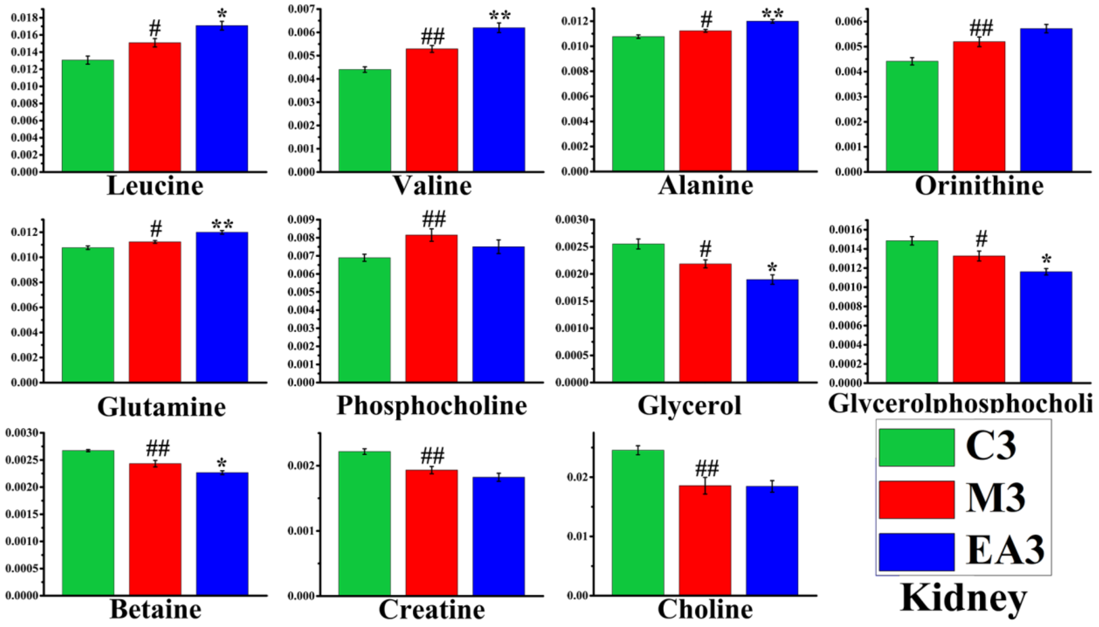

Fig.S12 Relative abundance (mean ± S.D.) of characteristic metabolites from kidney tissues of rats in C3, M3 and EA3 group.

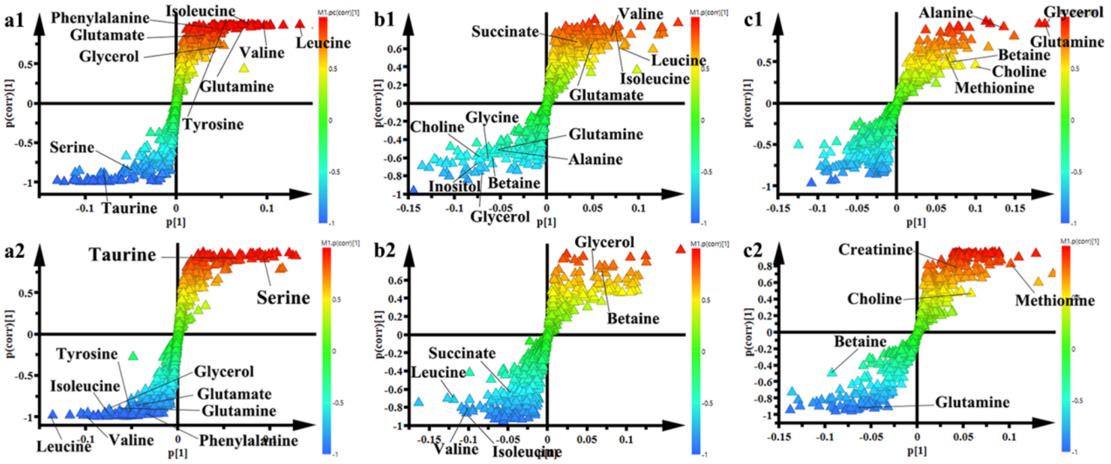

Fig.S13 Corresponding S-plots from stomach of rats in M1 and EA1 group (A); stomach of rats in M2 and EA2 group (B1); stomach of rats in M3 and EA3 group (C1); liver of rats in M1 and EA1 group (A2); liver of rats in M2 and EA2 group (B2); liver of rats in M3 and EA3 group (C2); kidney of rats in M1 and EA1 group (A3); kidney of rats in M2 and EA2 group (B3); kidney of rats in M3 and EA3 group (C3).

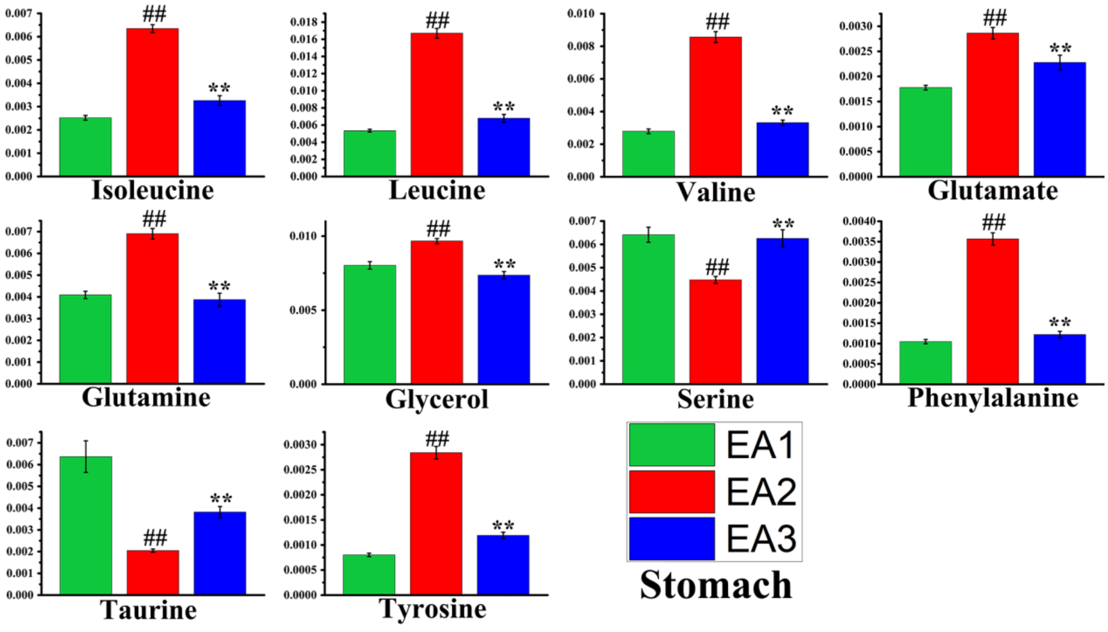

Fig.S14 Relative abundance (mean ± S.D.) of characteristic metabolites from gastric tissues of rats in EA1, EA2 and EA3 group.

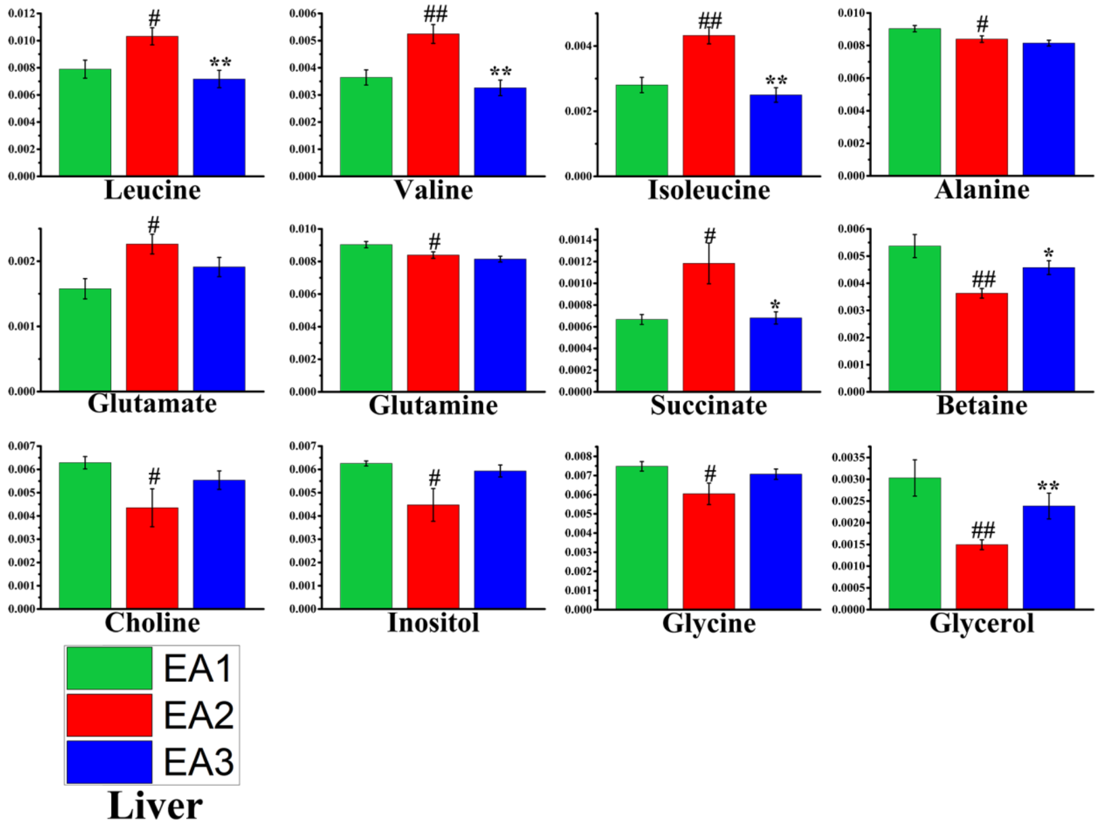

Fig.S15 Relative abundance (mean ± S.D.) of characteristic metabolites from liver tissues of rats in EA1, EA2 and EA3 group.

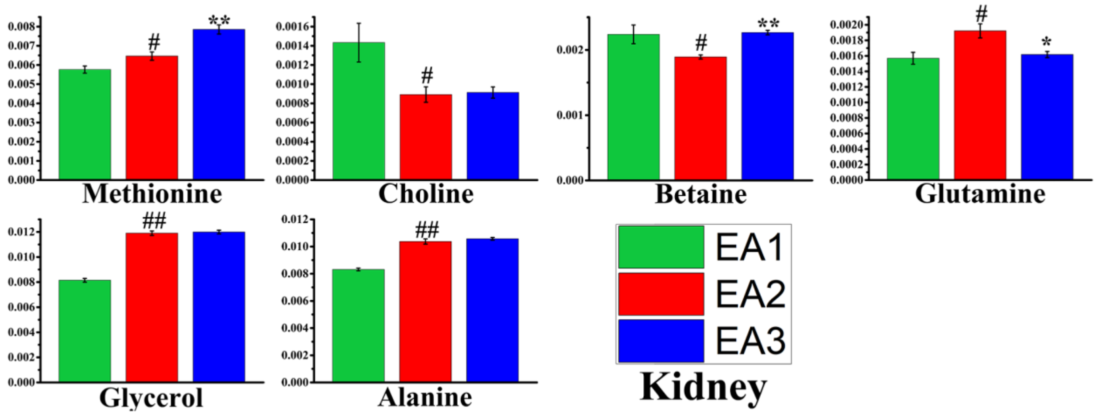

Fig.S16 Relative abundance (mean ± S.D.) of characteristic metabolites from kidney tissues of rats in EA1, EA2 and EA3 group.

Table S1 Peak attribution of the main marked metabolites in ^{1}H-NMR spectra of stomach sample

NO.	Metabolites	δ1H/ppm	Moieties
1	Isoleucine	0.94(t); 1.01(d)	δ-CH3; β-CH3
2	Leucine	0.96(t); 1.70(m)	CH3; CH2&γ-CH
3	Valine	0.99(d); 1.04(d)	γ-CH3; γ-CH3
4	3-Hydroxybutyrate	1.21(d)	γCH3 COSY
5	Methylmalonate	1.23(d)	CH3
6	Lactate	1.33(d); 4.11(q)	CH3; CH
7	Alanine	1.48(d); 3.78(q)	CH3; CH
8	Lysine	1.73(m); 1.91(m)	βCH2,δCH2
10	Acetate	1.92(s)	CH3
11	Glutamate	2.05(m)	β-CH
12	Glutamine	2.14(m)	β-CH2
14	Glutathione	2.17(m); 2.55(m)	β-CH2; γ-CH2
15	Succinate	2.41(s)	CH
17	Aspartate	2.69(dd);2.82(dd)	βCH2;βCH2
19	Methylguanidine	2.86(s) 3.38(s)	
21	Asparagine	2.88(dd);2.96(dd)	βCH2;βCH2
22	Creatine	3.04(s);3.94(s)	CH3,CH2
23	Creatinine	3.05(s)	CH3
24	Ethanolamine	3.15(t) 3.84(t)	CH2NH2; CH2OH
25	Choline	3.20(s);3.52(m);4.07(m)	CH3;N-CH2;O-CH2
26	Phosphocholine	3.22(s);3.59(m);4.17(m)	CH3;N-CH2;O-CH2
28	Glycerophosphocholine	3.23(s); 3.96(m)	CH3; CH&O-CH2
29	Acetylcholine	3.24(s)	
30	Betaine	3.27(s); 3.89(s)	CH3; CH2
32	Taurine	3.27(t);3.42(t)	S-CH2;N-CH2
33	Inosine	4.28(dd); 8.22(s)	CH(5); N-CH=N
34	Methanol	3.36(s)	28
36	Glycine	3.56(s)	CH2

37	Glycerol	3.57(m);3.62(m);3.79(m)	CH2; CH'2; CH
39	N,N-Dimethylglycine	2.92(s) 3.73(s)	
40	Serine	3.83(dd); 3.96(m)	CH; CH2
41	Phosphocreatine	3.93(s)	CH2
43	Adenosine monophosphate	4.03(m); 4.37(m)	O-CH2; CH; 66.92
44	Inosine	4.28(dd); 8.22(s)	CH(5); N-CH=N
45	Adenosine	4.30(dd);8.26(s); 8.35(s)	CH(5); N-CH=N
46	β-Glucose	4.64(d)	
47	α-Glucose	5.24(d)	
48	Allantoin	5.39(s)	CH
49	Uracil	5.80(d); 7.53(d) CH(5)	CH(6)
50	Uridine	5.90(d); 7.87(d)	CH(10); CH(11)
51	NADP+	6.05(d);6.15(d)	CH(32); CH(2)
54	Tyrosine	6.89(d); 7.19(d)	m-CH; o-CH
58	Phenylalanine ;	7.33(d); 7.38(t)	β-CH´; o-CH; p-CH
59	Xanthine	7.93(s)	CH(2);CH(9)

s: singlet, d: doublet, t: triplet, q: quartet, m: multiplet, dd: doublet of doublet.

Table S2 Peak attribution of the main marked metabolites in ^{1}H-NMR spectra of liver sample

**NO.**	**Metabolites**	**δ1H/ppm**	**Moieties**
1	Isoleucine	0.94(t); 1.01(d)	δ-CH3; β-CH3
2	Leucine	0.96(t); 1.70(m)	CH3; CH2&γ-CH
3	Valine	0.99(d); 1.04(d)	γ-CH3; γ-CH´3
4	3-Hydroxybutyrate	1.21(d)	γCH3
6	Lactate	1.33(d); 4.11(q)	CH3; CH
7	Alanine	1.48(d); 3.78(q)	CH3; CH
8	Lysine	1.73(m), 3.02(t)	βCH2,δCH2
10	Acetate	1.92(s)	CH3
11	Glutamate	2.05(m)	β-CH
12	Glutamine	2.13(m),3.77(t)	βCH2,γCH2
14	Glutathione	2.16 (m),2.55 (m)	β-CH2; γ-CH2
15	Succinate	2.41(s)	CH
18	Dimethylamine	2.72 (s)	CH3
20	N-methylhydantoin	2.92 (s),4.08 (s)	CH3,CH2
24	Ethanolamine	3.13 (d)	CH2
25	Choline	3.20(s);3.52(m);4.07(m)	CH3;N-CH2;O-CH2
26	Phosphocholine	3.22(s);3.59(m);	CH3; N-CH2;
27	Phosphoethanolamine	3.23(t);	NCH2;
28	Glycerophosphocholine	3.68(m);	N-CH2&HO-CH2;
30	Betaine	3.27(s); 3.89(s)	CH3; CH2
33	Inositol	3.28(t);3.54(dd);	CH(2); CH(4, 6);
36	Glycine	3.56 (s)	CH2
37	Glycerol	3.64 (m)； 3.77 (m)	CH2； CH
38	Glycogen	3.40(m)	1-CH

42	Glucaric acid	3.95(t)	CH(8)
46	β-glucose	4.63 (d)	1-CH
47	α-glucose	5.23 (d)	1-CH
48	Allantoin	5.39(s)	CH
49	Uracil	5.80(d); 7.53(d)	CH(5); CH(6)
50	Uridine	5.91(d);7.87(d)	CH(2);CH(11)
52	Cytidine	6.06(d); 7.84(d)	CH(2); CH(11)
53	Fumarate	6.52(s)	CH
54	Tyrosine	6.89(d); 7.19(d)	m-CH; o-CH
56	Tryptophan	7.19(m);7.31(s);7.60(m);	CH(8);CH(6);CH(7)
57	Nicotinamide	7.59(dd);8.24(dd);8.72(dd); 8.94(s)	CH(5);CH(4);CH(6); CH(2)
60	Hypoxanthine	8.19(s); 8.21(s)	CH(2); CH(7)
61	Formate	8.46(s)	CH

s: singlet, d: doublet, t: triplet, q: quartet, m: multiplet, dd: doublet of doublet.

Table S3 Peak attribution of the main marked metabolites in ^{1}H-NMR spectra of kidney sample

**NO.**	**Metabolites**	**δ1H/ppm**	**Moieties**
1	Isoleucine	0.94(t)	δ-CH3
2	Leucine	0.96(t);1.70(m)	CH3;CH2&γ-CH
3	Valine	0.99(d);1.04(d)	γ-CH3;γ-CH´3
4	3-Hydroxybutyrate	1.21(d)	γCH3
6	Lactate	1.33(d); 4.11(q)	CH3; CH
7	Alanine	1.48(d); 3.78(q)	CH3;CH
8	Lysine	1.73(m), 3.02(t)	βCH2,δCH2
9	Ornithine	1.73(m)	δCH2
10	Acetate	1.92(s)	CH3
11	Glutamate	2.05(m)	β-CH
12	Glutamine	2.13(m);3.77(t)	βCH2;γCH2
13	Methionine	2.14(s) ;2.65(t)	γCH2;S-CH3
14	Glutathione	2.16 (m);2.55 (m)	β-CH2; γ-CH2
15	Succinate	2.41(s)	CH
16	Citrate (M)	2.54(d);2.67(d)	CH2;CH2
17	Aspartate	2.69(dd);2.82(dd)	βCH2;βCH2
18	Dimethylamine	2.72 (s)	CH3
21	Asparagine	2.88(dd);2.96(dd)	βCH2;βCH2
22	Creatine	3.04(s);3.94(s)	CH3,CH2
23	Creatinine	3.05(s);4.06(s)	CH3;CH2
24	Ethanolamine	3.13 (d)	CH2
25	Choline	3.20(s);3.52(m);4.07(m)	CH3;N-CH2;O-CH2
26	Phosphocholine	3.22(s);3.59(m)	CH3; N-CH2;
28	Glycerophosphocholine	3.68(m)	N-CH2&HO-CH2
30	Betaine	3.27(s); 3.89(s)	CH3; CH2
31	Trimethylamine-N-oxide	3.27(s)	CH3

32	Taurine	3.27(t);3.42(t)	S-CH2;N-CH2
33	Inositol	3.28(t);3.54(dd)	CH(2); CH(4, 6);
35	Scyllo-Inositol	3.37(s)	CH
36	Glycine	3.56 (s)	CH2
37	Glycerol	3.64 (m)；3.77 (m)	CH2;CH
45	Adenosine	4.45(t);6.10(d);8.25(s);8.35(s)	3-C'H;1-C'H;8-CH;2-CH
46	β-glucose	4.63 (d)	1-CH
47	α-glucose	5.23 (d)	1-CH
48	Allantoin	5.39(s)	CH
49	Uracil	5.80(d); 7.53(d)	CH(5); CH(6)
50	Uridine	5.91(d);7.87(d)	CH(2);CH(11)
52	Cytidine	6.06(d); 7.84(d)	CH(2); CH(11)
53	Fumarate	6.52(s)	CH
54	Tyrosine	6.89(d); 7.19(d)	m-CH; o-CH
55	Histidine	7.11(s), 7.92(s)	2-CH, 4-CH
57	Nicotinamide	7.59(dd);8.24(dd); 8.72(dd); 8.94(s)	CH(5);CH(4);CH(6); CH(2)
59	Xanthine	7.93(s)	CH
60	Hypoxanthine	8.19(s); 8.21(s)	CH(2); CH(7)
61	Formate	8.46(s)	CH

s: singlet, d: doublet, t: triplet, q: quartet, m: multiplet, dd: doublet of doublet.

Table S4 Relative content in control, model and treatment group

**Metabolites**		**Stomach**			**Liver**			**Kindey**		
		**1day**	**4days**	**7days**	**1day**	**4days**	**7days**	**1day**	**4days**	**7days**
**Isoleucine**	Control	0.00271	0.00374	N	0.00241	N	0.00157	0.00758	0.00106	N
	GU model	0.00222	0.00523	N	0.00174	N	0.00094	0.00548	0.00047	N
	EA	0.00204	0.00654	N	0.0028	N	0.00227	0.00781	0.00117	N
**Leucine**	Control	0.00719	0.01032	N	0.00501	N	0.00433	0.01477	0.00159	0.01306
	GU model	0.00591	0.01727	N	0.00388	N	0.00283	0.01106	0.00133	0.01508
	EA	0.00534	0.0167	N	0.00598	N	0.00555	0.01478	0.00172	0.01707
**Valine**	Control	0.00274	0.004	N	0.00316	N	0.00085	0.00819	N	0.0044
	GU model	0.00221	0.00532	N	0.00248	N	0.00052	0.00692	N	0.00529
	EA	0.00193	0.00706	N	0.00364	N	0.00104	0.00817	N	0.0062
**Glycerol**	Control	0.00644	0.01694	0.00687	0.01484	0.00772	0.00639	0.00529	0.01065	0.00255
	GU model	0.00581	0.01067	0.00798	0.01665	0.01154	0.00892	0.00639	0.01143	0.00219
	EA	0.00617	0.01712	0.00787	0.01456	0.00746	0.00585	0.0053	0.01037	0.0019
**Glutamine**	Control	0.00501	N	N	0.01484	0.00443	0.0141	0.00682	0.00399	0.01076
	GU model	0.00413	N	N	0.01665	0.00609	0.0164	0.00541	0.00332	0.01123
	EA	0.00409	N	N	0.01456	0.00501	0.01501	0.00609	0.00439	0.01199
**Glutamate**	Control	0.00398	N	N	0.00159	N	0.00147	N	N	N
	GU model	0.00364	N	N	0.0013	N	0.00096	N	N	N
	EA	0.00372	N	N	0.00158	N	0.00157	N	N	N

**Tyrosine**	Control	0.00124	0.0018	N	0.00095	N	N	N	N	N
	GU model	0.00100	0.00276	N	0.00070	N	N	N	N	N
	EA	0.00090	0.00284	N	0.00102	N	N	N	N	N
**Choline**	Control	N	N	N	0.00646	0.00655	N	0.00486	0.00319	0.02454
	GU model	N	N	N	0.00716	0.00345	N	0.00595	0.00395	0.01856
	EA	N	N	N	0.00629	0.00435	N	0.00516	0.00309	0.01844
**Alanine**	Control	N	N	N	0.01484	0.01099	0.0141	0.01567	0.00241	0.01076
	GU model	N	N	N	0.01665	0.01315	0.0164	0.01353	0.00191	0.01123
	EA	N	N	N	0.01456	0.01108	0.01501	0.01676	0.00255	0.01199
**Glycine**	Control	N	N	N	0.01141	0.00621	N	0.00334	0.01987	N
	GU model	N	N	N	0.01238	0.00958	N	0.00461	0.02201	N
	EA	N	N	N	0.01113	0.00658	N	0.00385	0.0199	N
**Betaine**	Control	N	N	N	0.0051	0.00215	0.00462	0.00197	0.00249	0.00267
	GU model	N	N	N	0.00639	0.00321	0.00801	0.00243	0.00289	0.00243
	EA	N	N	N	0.00537	0.00202	0.00445	0.00216	0.00223	0.00227
**Serine**	Control	0.00421	0.00355	0.00498	N	N	N	N	N	N
	GU model	0.00496	0.00434	0.00592	N	N	N	N	N	N
	EA	0.00593	0.00542	0.00626	N	N	N	N	N	N
**Taurine**	Control	0.00085	0.00368	N	N	N	N	N	N	N
	GU model	0.0016	0.00164	N	N	N	N	N	N	N
	EA	0.00195	0.00205	N	N	N	N	N	N	N
**Phenylalanine**	Control	0.00157	0.00206	N	N	N	N	N	N	N
	GU model	0.00117	0.00357	N	N	N	N	N	N	N
	EA	0.00105	0.00357	N	N	N	N	N	N	N
**Inositol**	Control	N	N	N	0.01141	N	N	N	N	N
	GU model	N	N	N	0.01238	N	N	N	N	N
	EA	N	N	N	0.01113	N	N	N	N	N
**Succinate**	Control	N	N	N	0.00152	0.00079	N	N	N	N
	GU model	N	N	N	0.00092	0.00128	N	N	N	N
	EA	N	N	N	0.00112	0.00118	N	N	N	N
**Methionine**	Control	N	N	N	N	N	N	0.00682	0.00096	N
	GU model	N	N	N	N	N	N	0.00541	0.00073	N
	EA	N	N	N	N	N	N	0.00609	0.00107	N
**Glycerophosphocholine**	Control	N	N	N	N	N	N	0.00132	0.00508	0.00148
	GU model	N	N	N	N	N	N	0.00163	0.00537	0.00133
	EA	N	N	N	N	N	N	0.00134	0.00495	0.00116
**Ethanolamine**	Control	N	N	N	N	N	N	0.00243	0.00106	N
	GU model	N	N	N	N	N	N	0.00147	0.00047	N
	EA	N	N	N	N	N	N	0.00169	0.00131	N
**Creatine**	Control	N	N	N	N	N	N	0.00405	0.00378	0.00222
	GU model	N	N	N	N	N	N	0.00693	0.00427	0.00193
	EA	N	N	N	N	N	N	0.00483	0.00363	0.00182
**Phosphorylcho**	Control	N	N	N	N	N	N	0.00591	0.00445	0.00689
	GU model	N	N	N	N	N	N	0.00706	0.00487	0.00815

**line**	EA	N	N	N	N	N	N	0.00614	0.00426	0.0075
**Ornithin**	Control	N	N	N	N	N	N	0.00656	N	0.00441
	GU model	N	N	N	N	N	N	0.00515	N	0.00519
	EA	N	N	N	N	N	N	0.00631	N	0.00572

Red color: content increase, blue color: content decrease, N: no statistical significance.

Table S5 Relative content in different treatment group over time

**Metabolites**	**Stomach**			**Liver**			**Kindey**		
	**EA1**	**EA2**	**EA3**	**EA1**	**EA2**	**EA3**	**EA1**	**EA2**	**EA3**
Isoleucine	0.00252	0.00635	0.00326	0.0028	0.00432	0.0025	N	N	N
Leucine	0.00534	0.0167	0.00679	0.00789	0.01032	0.00717	N	N	N
Valine	0.00279	0.00857	0.00331	0.00364	0.00524	0.00326	N	N	N
Alanine	N	N	N	0.00904	0.00839	0.00815	0.00831	0.01037	0.01057
Betaine	N	N	N	0.00537	0.00363	0.00458	0.00224	0.00189	0.00227
Choline	N	N	N	0.00629	0.00435	0.00554	0.00143	0.00089	0.00091
Glutamate	0.00178	0.00286	0.00228	0.00158	0.00226	0.00191	N	N	N
Glutamine	0.00409	0.0069	0.00387	0.00904	0.00839	0.00815	0.00157	0.00192	0.00162
Glycerol	0.00803	0.00967	0.00736	0.00303	0.00149	0.00238	0.00814	0.0119	0.01199
Glycine	N	N	N	0.00748	0.00605	0.00707	N	N	N
Inositol	N	N	N	0.00626	0.00447	0.00593	N	N	N
Methionine	N	N	N	N	N	N	0.00576	0.00646	0.00785
Phenylalanine	0.00105	0.00357	0.00122	N	N	N	N	N	N
Serine	0.00641	0.00448	0.00626	N	N	N	N	N	N
Succinate	N	N	N	0.00067	0.00118	0.00068	N	N	N
Taurine	0.00637	0.00205	0.00382	N	N	N	N	N	N
Tyrosine	0.00080	0.00284	0.00119	N	N	N	N	N	N

Red color: content increase, blue color: content decrease, N: no statistical significance.

Table S6 p (CV-ANOVA) indicating the model quality of OPLS-DA

OPLS-DA model			p(CV-ANOVA)		
			stomach	liver	kidney
GU model vs.	1d	control	0.0350044	0.150404	0.00910267
		EA	0.392812	0.0389342	0.0802677
	4d	control	0.342957	0.0836458	0.0759741
		EA	0.000071	0.0291931	0.00442573
	7d	control	0.439592	0.205449	0.0171049
		EA	0.339974	0.0188815	0.0460066
